# Anthropometric Cut-Off Values for Detecting the Presence of Metabolic Syndrome and Its Multiple Components among Adults in Vietnam: The Role of Novel Indices

**DOI:** 10.3390/nu14194024

**Published:** 2022-09-28

**Authors:** Anh Kim Dang, Mai Tuyet Truong, Huong Thi Le, Khan Cong Nguyen, Mai Bach Le, Lam Thi Nguyen, Khanh Nam Do, Lan Huong Thi Nguyen, Abdullah A. Mamun, Dung Phung, Phong K. Thai

**Affiliations:** 1 Queensland Alliance for Environmental Health Sciences (QAEHS), The University of Queensland, Woolloongabba, QLD 4102, Australia; 2 Institute for Preventive Medicine and Public Health, Hanoi Medical University, Hanoi 100000, Vietnam; 3 National Institute of Nutrition, Hanoi 100000, Vietnam; 4 Poche Centre for Indigenous Health, The University of Queensland, Indooroopilly, QLD 4068, Australia; 5 ARC Centre of Excellence for Children and Families over the Life Course (The Life Course Centre), The University of Queensland, Indooroopilly, QLD 4068, Australia; 6 School of Public Health, Faculty of Medicine, The University of Queensland, Herston, QLD 4006, Australia

**Keywords:** metabolic syndrome, obesity, anthropometry, Vietnam

## Abstract

Recent studies have shown that using international guidelines to diagnose metabolic syndrome (MetS) may underestimate its prevalence in different Asian populations. This study aims to determine the validity of anthropometric indicators and appropriate cut-off values to predict MetS for Vietnamese adults. We analyzed data on 4701 adults across four regions of Vietnam. Four conventional and five novel anthropometric indexes were calculated. The area under a receiver operating characteristic (ROC) curve (AUC) and Youden’s J statistic were applied to evaluate the diagnostic ability and optimal cut-off values. Regardless of diagnostic criteria and gender, Abdominal volume index (AVI), Body roundness index (BRI), and Waist-height ratio (WHtR) had the highest AUC values, followed by Body mass index (BMI) and Waist-hip ratio (WHR). However, it was seen that differences among the AUC values of most indices were minor. In men, using International Diabetes Federation (IDF) criteria, the threshold of indices was 3.86 for BRI, 16.20 for AVI, 0.53 for WHtR, 22.40 for BMI, and 0.90 for WHR. In women, the threshold for these figures were 3.60, 12.80, 0.51, 23.58, and 0.85, respectively. It is recommended that health personnel in Vietnam should apply appropriate thresholds of anthropometry, which are lower than current international guidelines, for MetS screening to avoid under-diagnosis.

## 1. Introduction

Metabolic syndrome (MetS) is a constellation of several clinical features, including dyslipidemia, hypertension, dysregulated glucose homeostasis, and abdominal obesity [1]. MetS has been identified and attracted much attention over the last decades because of its close association with obesity, diabetes, and cardiovascular complications [2,3]. Effective techniques to identify MetS in the population play an integral part in applying early interventions to prevent adverse health outcomes caused by MetS, such as obesity and diabetes. Previous studies emphasized using anthropometric measurements to identify nutritional status and MetS [4]. This method’s main advantages cover not only past exposures, current processes, or future events but also non-invasiveness, standardized techniques, easy measuring, and interpretation [5]. From the community perspective, anthropometric indices are helpful for self-assessment of the chronic imbalance of dietary intake, which could lead to MetS [6]. From the clinical point of view, body indices are practical to evaluate the nutritional status of patients and monitor malnutrition in all subjects [6]. Using such simple measurements to identify MetS is even more critical in low-income countries with limited resources for public health and healthcare.

Several anthropometric indicators can be used to predict the presence of MetS. Body mass index (BMI), which is based on the height and weight of individuals, is reported to help estimate obesity and MetS because of its simplicity, low cost, ability to apply on a large scale, and comparability to different populations [7]. However, because BMI solely relies on height and weight, it limits the ability to distinguish the excess condition of fat mass or lean body mass, as well as does not provide information on the fat distribution of individuals. To overcome such disadvantages, body fat percentage (%BF), WC, waist-to-hip ratio (WHR), and waist-to-height ratio (WHtR) were suggested to be used to assess total body fat and fat distribution [6]. Nevertheless, WC values alone are inaccurate because higher people may have greater circumferences and differ between ethnic groups [8]. Concerning WHtR, a previous study revealed higher predictive values of WHtR in cardiovascular risk compared to a combination of WC and BMI [9]. In addition, WHR has been reported to have better discriminative power of MetS than BMI [10].

State-of-the-art anthropometric indicators combining measurements were utilized to define the MetS among the study population. A body shape index (ABSI) was developed in 2012 by Krakauer et al., and its value positively correlated with the abdominal accumulation of adipose tissue [11]. In 2013, Thomas et al. published a new body index, the Body roundness index (BRI), which modeled the body shape as an ellipse or an oval and the relationship between height and body girth [12]. Compared to other traditional and novel anthropometric indices, BRI showed a better prediction of MetS [13]. Abdominal Volume Index (AVI), which used both waist and hip circumferences in the formula, was a reliable index for evaluating obesity and is closely associated with impaired glucose tolerance and diabetes [14]. Body adiposity index (BAI) was proposed by Bergman et al. in 2011 as an alternative parameter based on the ratio between hip circumference (HC) and height [15]. As BAI can directly evaluate fat mass without general characteristics such as gender and age, it has been widely used recently to estimate adiposity and risk for cardio-metabolic diseases [16]. Conicity Index (CI) advantages over WHR because it includes WC, weight, and height in its calculation without HC and is a simple indicator to assess fat distribution [14].

A growing body of evidence suggests that Asian populations have lower cut-off values of anthropometric indicators for MetS than Europeans and international standards [2,17,18,19]. It is hence essential to develop new practical values to determine MetS for an individual country, including Vietnam, to avoid underestimation of MetS prevalence. However, based on our knowledge, none of the published studies examines the use of anthropometric indices of Vietnamese adults to predict MetS. Currently, physicians and health personnel in Vietnam still apply international guidelines in detecting MetS. Our findings will provide useful evidence for screening MetS during clinical examination when not all components of MetS are evaluated, such as blood tests for dyslipidemia indicators. Therefore, in this study, we aim to determine discriminative power and cutoff values of anthropometry (BMI, %BF, WHR, WHtR, AVI, BRI, ABSI, BAI, CI) as predictors of MetS specific for the Vietnamese adult population using data from a large national study.

## 2. Materials and Methods

### 2.1. Study Design and Subjects

A cross-sectional study was carried out from June 2007 to July 2008 in four different regions of Vietnam, which consisted of urban (Hanoi City and Ho Chi Minh City), rural (Northern Delta and Mekong River Delta), mountainous (Northern mountainous region and Central Highlands) and coastal areas (Coastal areas in North Central and South Central). Table 1 describes the name of cities and areas of study locations.

Participants were selected among Vietnamese adults aged from 25 to 74 years old. People who had the following criteria were excluded from the study (1) Having physical disabilities; (2) Suffering from an acute disease at the time of studying; (3) Having dementia, deaf, weak; (4) Being a pregnant woman, or breastfeeding mothers within 12 months after giving birth. Participants’ health information was collected based on medical records via self-reported and commune health stations.

### 2.2. Sample Size and Sampling Technique

This study applied the sample size and sampling technique based on a previous prevalence study of MetS [20], illustrated by the following formula.

n=Z2(1−α/2)p×(1−p)d^2

In which, “*n*” presents total number of subjects in each study region; “*Z*” = 1.96 with 95% confidence interval; “*d*” = 0.03 illustrating precision (3%); “*p*” = 0.131, percentage of people having MetS based on a previous study [21]. Therefore, the sample size for each region was 600 adults. Due to the cross-sectional study design using cluster sampling, we used a design effect of two (DE = 2) and doubled the sample size (600 × 2 = 1200). The total sample size in four regions was 4800 (1200 × 4 = 4800). The actual sample size was 4701, with a response rate of 97.9%. A simple two-stage cluster sampling technique was applied to recruit study participants, in which we purposely selected twenty clusters of communes/wards in each study area, and participants were randomly chosen in each cluster.

Data of this study were retrieved from a report of the National Institute of Nutrition (NIN) project for the Government of Vietnam (No. KC.10.05/06-10), which aimed to assess lipid nutritional disorders in adults in the community and several preventive interventions. Our utilization of data provides a better understanding of using anthropometry thresholds to predict MetS in Vietnamese adults and plays a fundamental part in future studies focusing on how nutritional pattern changes could affect this association.

### 2.3. Measurements and Instruments

#### 2.3.1. Anthropometric Measurements and Biochemical Test

An interviewer-administered questionnaire was carried out to collect socio-economic characteristics and health risk behavior of participants, which consisted of age, gender, living areas, ethnicity, smoking, alcohol use, and salty eating habits.

Blood pressure and anthropometric indicators, including height, weight, hip, waist circumstance, and body fat percentage, were measured by well-trained staff at commune health stations using standard instruments.

- We used a SECA scale with an accuracy of 0.1 kilograms (kg) to assess body weight. The body weight was reported in kg with a decimal. Participants’ scale was measured in the morning without eating anything and after urinating. Participants stood in the middle of the scale; their eyes looked straight, and their weight was evenly distributed on both legs. The scale was placed in a stable and flat position.
- A microtoise gauge height was utilized to estimate vertical height with an accuracy of 1 millimeter (mm). Height was recorded in centimeters (cm) with a decimal. Participants took off their shoes and stood with his/her back to the ruler. Heels, legs, buttocks, shoulders, and head stayed in a straight line against the vertical ruler, while eyes looked straight in a horizontal line. Two hands hung along his/her sides. A researcher pulled the top of the ruler from the top-down and read the result when the ruler’s top touched close to the crown of the head.
- An elastic band estimated waist and hip circumstances (WC and HC), and the results were reported in centimeters (cm) with a decimal. In this study, to measure the WC, we determined the lowest point of the lower rib and the upper edge of the iliac crest and then took the midpoint of these two positions. In addition, the fullest part of the buttock got the measure of the HC.
- Body fat percentage was calculated by a Japanese OMRON bio-electrometer, which was recommended if there was a presence of technical error, such as using a 3-site skinfold [22].
- We applied a mercury sphygmomanometer to assess the blood pressure of respondents two times for two minutes apart. The final result was calculated as the average of the two measurements. If the parameters between the two times differed by more than ten millimeters of mercury (mmHg), the third measurement must be repeated.

Nurses from the commune health stations took the blood of participants for biochemical blood tests, which covered fasting blood glucose, insulin, triglycerides, low-density lipoprotein cholesterol (LDL-C), and high-density lipoprotein cholesterol (HDL-C) to determine dyslipidaemias and metabolic syndrome.

Participants rested for at least 10 min and were taken 5 mL of fasting venous blood. Participants were required to fast for at least 10 h, preferably overnight, but not more than 16 h. Participants who had fever were asked to take a blood test one week after.

The blood test was done in the field by the Acucheck machine according to the colorimetric method. We put 5 mL of blood into a test tube containing pre-made plastic particles. Blood samples were centrifuged within 10 minutes in the field to extract serum and plasma. Collected specimens were stored under refrigeration from +2 °C to +8 °C in the field, during transport, and at −20 °C until analysis. The criteria and methods of analysis were described as follows:

- Total cholesterol was quantified by the CHOD-PAP method, which used an enzyme (enzymatic colorimetric), cholesterol oxidase phenazon amino peroxidase.
- Serum triglycerides were measured using the colorimetric GPO-PAP method (Glycerol phosphate oxidase phenazon amino oxidase).
- Serum HDL-C was assessed by the method of precipitation of LDL-C, very-low-density lipoprotein (VLDL) cholesterol, and chylomicrons.

#### 2.3.2. Anthropometric Indices and Metabolic Syndrome Definition

To detect MetS, the following anthropometric indices were considered: Body mass index [BMI], Body fat percentage [%BF], Waist-height ratio [WHtR], Waist-hip ratio [WHR]. In addition, several new parameters had been proposed including Abdominal Volume Index [AVI], Body roundness index [BRI], A body shape index [ABSI], Conicity index [CI], Body adiposity index [BAI]. Equations of all indices were presented below:

- Body mass index (BMI) = Weight (kg)/Height^2^ (m)
- Waist-height ratio [WHtR] = WC (cm)/Height (cm)
- Waist-hip ratio [WHR] = WC (cm)/HC (cm)
- Abdominal volume index [AVI] = (2 × WC^2^ (cm) + 0.7 × (WC-HC)^2^ (cm))/1000
- Body roundness index [BRI] = 364.2 − 365.5 ×$\;\sqrt{1-\left(\frac{{\left(\frac{\mathrm{WC}\left(m\right)}{2\pi }\right)}^{2}}{{\left(0.5*\mathrm{height}\left(m\right)\right)}^{2}}\right)}$
- A body shape index [ABSI] = WC (m)/(BMI^2/3^ × Height^1/2^ (m))
- Conicity index [CI] = WC (m)/(0.109 ×$\sqrt{\frac{\mathrm{Weight}\;\left(\mathrm{kg}\right)}{\mathrm{Height}\;\left(m\right)}}$)
- Body adiposity index [BAI] = (HC (cm)/Height^1.5^(m)) − 18

The criteria of the National Cholesterol Education Program, Adult Treatment Panel III (NCEP ATP III), and International Diabetes Federation (IDF) with modified cut-off values of central obesity for Asians [23] were used to determine the metabolism disorder of participants. Regarding NCEP ATP III, having 3 or more signs listed below was diagnosed with MetS [24]:

- Abdominal fat in which waist circumference was ≥90 cm for males and ≥80 cm for females
- High triglycerides (≥1.7 mmol/L or ≥150 mg/dL)
- Low HDL-C (<40 mg/dL (<1.03 mmol/L) for males, <50 mg/dL (<1.29 mmol/L) for females)
- Elevated blood pressure which was presented as systolic hypertension (SBP ≥ 130 mmHg) or diastolic hypertension (DBP ≥ 85mmHg)
- High fasting blood glucose (≥5.6 mmol/l or ≥100 mg/dL)

While visceral obesity is not a prerequisite factor of NCEP ATP III, it is considered a mandatory IDF criterion, with at least two out of the remaining four criteria [24].

### 2.4. Data Analysis

Data were entered using Epidata 3.2 software and analyzed by STATA 15 (StataCorp, College Station, TX, USA). In this study, to test the differences in general characteristics and anthropometric indices by gender, we utilized the Chi^2^ test for categorical variables and the Wilcoxon-Mann-Whitney test for continuous variables. To assess the performance of a diagnostic test (overall power, sensitivity, and specificity), the multivariate receiver-operating characteristic (ROC) curve was applied to men and women separately within the range of values of predictor variables. The area under the curve (AUC) evaluated the diagnostic ability to discriminate the diseased and healthy populations, allowing researchers to estimate the accuracy of two or more diagnostic tests. ROC analysis and the AUC comparison method are popular techniques to measure the performance of medical diagnostic biomarkers [25], which have been used extensively in clinical epidemiology [26]. In addition, we used Youden’s J statistic to find the optimal cut-off value of all anthropometric indicators, which was based on the principle of the equation: Jmax. = Sensitivity + Specificity − 1. These cut-off points optimize the differentiating ability of anthropometric parameters in detecting MetS by the equal weight of sensitivity and specificity, which maximized the value of Youden’s J statistic [27]. To compare the differences in area under the ROC curve (AUC) of anthropometry indices, we applied the “roccomp” command in Stata [28].

### 2.5. Ethical Statement

Participants received written informed consent and were explained the study’s purposes prior to the research. Participants can refuse or withdraw from the research at any time. The Vietnamese National Institute of Nutrition ethics committee approved the ethics of the study (No. 536/QĐ-VDD).

## 3. Results

### 3.1. General Information of Participants

Table 2 presents the general characteristics of the study population. People who experienced MetS had significantly greater weight, height, and waist/hip circumference than those without MetS. Regarding anthropometric indicators, BMI, WHR, %BF, AVI, BRI, and BAI were significantly higher in participants having MetS. Metabolic component profiles also differed between the two groups. While MetS people experienced higher levels of blood pressure, fasting blood glucose, triglycerides, total cholesterol, and LDL cholesterol, we found that HDL cholesterol values of them were lower than that of people without MetS.

The socio-economic characteristics of participants are shown in Table S1 (Supplementary Material). Male participants accounted for 48.4% (2277 subjects) of the total. The mean age of respondents was 49.6 (SD = 14.1) and this figure was higher in the group with MetS than the group without MetS. The percentage of having salty eating habits was significantly higher in the group having MetS than in the without-MetS group (*p* < 0.01).

### 3.2. ROC Curve Analysis and Cut-Off Points Estimation

Table 3 and Table 4 depict the cut-off points of anthropometric indices for the prediction of MetS components in men and women respectively. Using IDF definition, BMI cut-off values to determine MetS in men was 22.40 (AUC = 0.93) and in women was 23.58 (AUC= 0.90). In addition, WHR, AVI and BRI also had high discriminatory power for detecting MetS in both genders, with cut-off values of WHR = 0.9 (AUC = 0.94), AVI = 16.2 (AUC = 0.99), BRI = 3.86 (AUC = 0.97) in males and WHR = 0.85 (AUC = 0.90), AVI = 12.8 (AUC = 0.97), BRI = 3.60 (AUC = 0.96) in females.

Regarding other metabolic components in men (Table 3), to evaluate abnormal triglycerides concentration, the largest AUC was for the AVI (0.70 and cut-off value = 11.7) and the WHR (0.68 and cut-off value = 0.85). To diagnose MetS using the ATP III definition, the AVI had a higher AUC value (0.77) than BMI (0.74) and WHR (0.73). Besides, the WHtR cut-off value of 0.50 (AUC = 0.76) and BRI cut-off value of 3.37 (AUC = 0.76) had high discriminatory power in detecting MetS.

In women (Table 4), early diagnosis of elevated triglycerides was determined based on WHtR, AVI and BRI with cut-off values of 0.48 (AUC = 0.72), 10.71 (AUC = 0.72) and 2.94 (AUC = 0.72), respectively. Unlike men, to evaluate MetS by the ATP III, BMI with a cut-off value of 22.31 (AUC = 0.83) had better discriminatory power than the WHR value. The optimal cut-off point for the MetS identification of WHtR and BRI were 0.49 (AUC = 0.84) and 3.14 (AUC = 0.84), respectively

**Figure 1:**
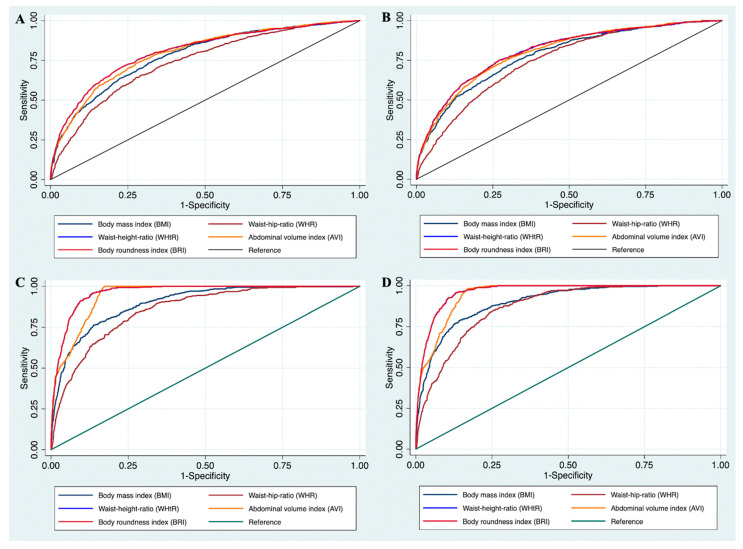
The receiver operating characteristic (ROC) curves for Body mass index (BMI), Waist–hip ratio (WHR), Waist–height ratio (WHtR), Abdominal volume index (AVI), and Body roundness index (BRI) values to detect metabolic syndrome by 1. National Cholesterol Education Program, Adult Treatment Panel III (NCEP ATP III) in males (**A**) and females (**B**); 2. International Diabetes Federation (IDF) in males (**C**) and females (**D**). *p*-values in four models <0.01 which reflect the differences in area under the ROC curve are statistically significant.

Figure 1 shows the ROC curves to determine the appropriate cut-off values of BMI, WHR, WHtR, AVI, and BRI for detecting the presence of MetS by NCEP ATP III and IDF in males and females. Using NCEP ATP III criteria, the cut-off values of 3.37 in men and 3.14 in women (for BRI), 0.76 in men and 0.49 in women (for WHtR), 12.94 in men and 12.37 in women (for AVI) had high AUC in diagnosis MetS in both genders. The cut-off values to detect MetS by IDF in males were 0.53 for WHtR, 16.20 for AVI, and 3.86 for BRI. These figures for females were 0.51 for WHtR, 12.80 for AVI, and 3.60 for BRI, respectively.

Table 5 illustrates the logistic regression models in which each anthropometric indices was a primary predictor variable for the MetS diagnosis by NCEP ATP III in both sexes. Adjusting for age, living areas, ethnicity, smoking, alcohol use, and salty eating habits, significant relationships were found between metabolic abnormalities and all anthropometric indicators. WHtR, AVI, and BRI had the strongest association with MetS in men and women compared to other parameters. In addition, people with BMI and %BF cut-off values of 21.30 and 23.1% in men or 22.31 and 30.90% in women were significantly more likely to experience MetS using ATP III criteria.

## 4. Discussion

Our research is one of the first studies providing crucial evidence in identifying people at high risk of MetS based on anthropometric indices in Vietnam. These results are useful in practical clinical examination since MetS may otherwise be overlooked due to an absence of risk factors, for instance, low level of HDL-C or high LDL-C level. Cut-off values of anthropometric indicators to predict MetS differed depending on gender and components of metabolic abnormality. All indicators had higher AUC values to determine MetS by IDF rather than by NCEP ATP III. Regardless of diagnosis criteria and gender, WHtR and BRI had the largest AUC, followed by AVI and BMI. Regarding detecting high triglycerides, AVI had the highest AUC; however, the predictive ability was moderate accuracy.

According to our results, BRI and WHtR had the highest AUC in predicting MetS using IDF criteria, with the cut-off values of 3.86 and 0.53 in men; 3.60 and 0.51 in women, respectively. BRI is developed to advance WC, which combines height, and WC and captures the body shape as an ellipse or an oval to predict %BF and total % visceral adipose tissue [15]. WHtR was also reported as one of the useful anthropometric indexes in screening cardiovascular diseases by deeming the simultaneous impact of height and WC on body fat composition [29]. Our findings are consistent with previous studies, which revealed the high discriminatory power of BRI and WHtR (WHtR cut-off values of approximately 0.5 in both genders) in detecting MetS [18,30,31,32]. However, the cut-off points of BRI and WHtR in this study are lower than that of the European population (BRI of 4.612 in men and 4.934 in women; WHtR of 0.549 in men and 0.532 in women) to define at least one MetS component [5]. A previous meta-analysis showed similar results to our study, which indicated that pooled AUC values of BRI and WHtR in predicting MetS were 0.77 (0.73–0.80) and 0.78 (0.72–0.83), respectively, in all subjects, higher than that of WHR and BAI [13]. This can be explained by the advantages of BRI in enhancing the predictive power by reflecting body fat and visceral adipose tissue associated with the presence of MetS [12]. Regarding WHtR, among the Asian population, WHtR performed to be superior to BMI in predicting severe outcomes related to cardiometabolic risk [33,34]. A previous study suggested that WHtR attracted interest as it was one of the best simple anthropometric indices to screen several cardiovascular risk factors and severe outcome conditions [35].

We also applied AVI, one of the new anthropometric indices deliberating adipose tissue in viscera and abdominal organs, to predict MetS among healthy Vietnamese adults. In this study, AVI showed a high discriminatory power for detecting MetS in both genders, with cut-off points of 16.20 in men and 12.80 in women using IDF criteria. This result is in agreement with studies conducted among the adolescent population, which reveal that AVI had a high discriminatory capacity (AUC > 0.8) for detecting MetS compared to other anthropometric indices [30,36]. The high predictive ability of AVI on the development of MetS can be explained by the close relationship between fatty tissue distribution and metabolisms in the organs, such as visceral obesity, insulin resistance, and dyslipidemia [36]. Previous research also highlights that higher AVI values were strongly associated with elevated glucose, impaired glucose tolerance, and type 2 diabetes [37].

We found that BMI and WHR had higher discriminatory power and cut-off values using IDF criteria than NCEP ATP III selection. This finding is similar to the results of a study carried out on Spanish adolescents, which showed that BMI had a high discriminatory capacity for MetS (AUC > 0.8) only when utilizing the IDF criteria [38]. The difference can be explained that abdominal obesity is a prerequisite for MetS diagnosis based on the definition of IDF or using IDF definition to detect MetS would include more people with central obesity [39]. However, the limitation of BMI is considering neither differing lean mass and fat mass nor body fat distribution. The BMI cut-off values to detect MetS in our study are lower than the obesity cut-off point for the Asia-Pacific region defined by WHO (BMI ≥ 25 kg/m^2^) [40]. Our finding is higher than the cut-off value for BMI in defining high blood pressure among the Vietnamese population (ranging from 20.5 to 21) [41]. Findings from our research are similar to those obtained in the Chinese population (2011), which shows that BMI and WHR cut-off points to determine MetS multiple risk factors were 22.85 kg/m^2^ and 0.87 (in males); 23.30 kg/m^2,^ and 0.85 (in females) respectively [18]. Similar to the results of our analysis, a study conducted on the Korean population (2014) concluded that the BMI cut-off value to predict MetS using NCEP ATP III was 24.2 kg/m^2^ in males and 25.3 kg/m^2^ in females [42]. Another study in Koreans (2011) using IDF criteria also presented similar findings to BMI and WHR threshold to our research (24.1 kg/m^2^, 0.9 in men and 24.0 kg/m^2^, 0.9 in women, respectively) [43]. In addition, another study of non-Asians revealed higher cut-off points than our results. Stanisław Głuszek recommended using BMI to estimate at least one MetS component in the European population, with a cut-off of 27.65 in men and 27.41 in women [5].

### 4.1. Implications

Several implications can be drawn from the research. Firstly, BRI, and AVI, novel anthropometry, were better than other indices in predicting MetS, regardless of diagnostic criteria and gender. Regarding clinical practice, we recommend the use of WHtR, BMI, and WHR as they have high accuracy while being simple, conveniently measuring in all clinical settings, and efficiently interpreting, especially using IDF criteria. In addition, BRI, AVI, and WHtR have moderate accuracy in defining high triglyceride conditions in both genders. As the thresholds determined by our study are lower than current guidelines, we suggest that physicians and health personnel consider using the new cut-off values of anthropometry to identify obesity-related health problems in the Vietnamese population. Finally, taking the place of measuring anthropometric indicators such as height, weight, WC, and HC should be promoted during periodic health checks in adults, instead of height and weight only.

### 4.2. Strengths and Limitations

The strength of our study is using updated anthropometric indices to predict the presence of MetS among Vietnamese adults and increasing the comparative ability of the study to other international research. A large number of participants were randomly recruited from four main regions of Vietnam, which may enhance the generalizability of this study. However, some limitations should be acknowledged. We did not analyze the comprehensive impacts of anatomical or functional changes in adipose tissue, eating habits, and individual physical activity, which are considered primary factors of MetS occurrence. A cross-sectional study design may limit the capacity to interpret the temporal relationship between indicators and MetS. Finally, we derived the threshold by querying the data of a project conducted in 2008 in Vietnam. The socio-demographic features and anthropometry profiles in the country could have changed significantly from this period until now. Therefore, we suggest further study to determine up-to-date cut-off values of anthropometric indices. A new study could also confirm the causal association between the change in anthropometry and the occurrence of MetS among adults.

## 5. Conclusions

In conclusion, we emphasize the advantages of using novel indices, BRI and AVI, in predicting the presence of MetS among Vietnamese adults. From the clinical perspective, WHtR had superior abilities, along with BMI, to determine MetS because of convenient measurement and easy interpretation. Health personnel should apply appropriate thresholds of anthropometry to define MetS for the Vietnamese, which are lower than current international guidelines.

## Figures and Tables

**Table 1:** Settings of the study.

Regions of Vietnam	City and Areas
Urban areas	Hanoi city and Ho Chi Minh city
Rural areas	Northern delta and Mekong River delta
Mountainous areas	Northern mountainous region and Central Highlands
Coastal areas	Coastal areas in North Central and South Central

**Table 2:** Baseline anthropometric and laboratory characteristics of participants by metabolic syndrome condition using National Cholesterol Education Program, Adult Treatment Panel III (NCEP ATP III) criteria.

	Having MetS		Without MetS		Total		*p*-Value
	Mean	SD	Mean	SD	Mean	SD	
MetS by NCEP ATP III (*n*, %)	1056	22.5	3645	77.5			
MetS by IDF (*n*, %)	468	10.0	4233	90.0			
Weight (kg)	57.5	10.4	51.3	8.5	52.7	9.3	<0.01 *
Height (cm)	156.0	8.1	157.0	7.7	156.8	7.8	<0.01 *
Waist circumference (WC, cm)	81.4	9.8	71.8	7.8	73.9	9.2	<0.01 *
Hip circumference (cm)	92.2	7.0	87.2	5.9	88.3	6.5	<0.01 *
Body mass index (kg/m^2^)	23.6	3.4	20.8	2.6	21.4	3.1	<0.01 *
Waist–hip ratio (WHR)	0.9	0.1	0.8	0.1	0.8	0.1	<0.01 *
Waist–height ratio (WHtR)	0.5	0.1	0.5	0.1	0.5	0.1	0.50 *
Body fat percentage (%)	29.8	6.5	23.6	6.5	25.0	7.0	<0.01 *
Systolic blood pressure (mmhg)	142.3	21.7	121.6	20.2	126.3	22.3	<0.01 *
Diastolic blood pressure (mmhg)	86.4	12.4	75.5	12.1	78.0	13.0	<0.01 *
Total cholesterol (mg/dL)	5.4	1.2	4.7	1.0	4.9	1.1	<0.01 *
HDL cholesterol (mg/dL)	1.0	0.2	1.3	0.3	1.2	0.3	<0.01 *
LDL cholesterol (mg/dL)	3.3	1.1	2.7	0.9	2.9	0.9	<0.01 *
Triglycerides (mg/dL)	2.7	1.6	1.4	0.9	1.7	1.2	<0.01 *
Fasting blood glucose (mg/dL)	6.0	2.0	4.9	0.9	5.2	1.3	<0.01 *
Abdominal Volume Index (AVI)	13.5	3.2	10.6	2.2	11.3	2.8	<0.01 *
Body roundness index (BRI)	3.9	1.2	2.6	0.8	2.9	1.1	<0.01 *
A body shape index (ABSI)	0.08	0.01	0.08	0.01	0.08	0.01	0.05 *
Body adiposity index (BAI)	29.5	4.5	26.4	3.6	27.1	4.0	<0.01 *
Conicity index (CI)	1.2	0.1	1.2	0.1	1.2	0.1	0.05 *

* Mann-Whitney U test.

**Table 3:** Areas under the curve (AUCs) and cut-off points for anthropometric indices for the prediction of MetS components in men.

MetS Components	Indices	AUC	Sensitivity	Specificity	Youden Index	Cut-Off Points
High triglycerides	BMI	0.69	0.63	0.68	0.31	21.3
	%BF	0.69	0.64	0.64	0.27	22.5
	WHR	0.68	0.63	0.65	0.28	0.85
	WHtR	0.70	0.56	0.75	0.31	0.48
	AVI	0.70	0.60	0.71	0.32	11.7
	BRI	0.70	0.56	0.75	0.31	3.00
	ABSI	0.60	0.65	0.51	0.17	0.08
	BAI	0.63	0.65	0.55	0.20	24.6
	CI	0.65	0.58	0.67	0.25	1.19
Low HDL-C	BMI	0.59	0.55	0.61	0.16	21.3
	%BF	0.59	0.37	0.78	0.15	25.80
	WHR	0.57	0.42	0.70	0.12	0.88
	WHtR	0.58	0.49	0.65	0.14	0.48
	AVI	0.59	0.37	0.78	0.15	13.2
	BRI	0.58	0.49	0.65	0.14	2.90
	ABSI	0.53	0.45	0.62	0.07	0.08
	BAI	0.57	0.60	0.52	0.12	24.70
	CI	0.55	0.47	0.64	0.11	1.20
Increased blood glucose	BMI	0.61	0.50	0.63	0.14	21.7
	%BF	0.62	0.56	0.63	0.19	23.6
	WHR	0.63	0.54	0.66	0.20	0.87
	WHtR	0.64	0.46	0.75	0.21	0.49
	AVI	0.64	0.52	0.69	0.21	12.3
	BRI	0.64	0.46	0.75	0.21	3.26
	ABSI	0.63	0.62	0.59	0.21	0.08
	BAI	0.60	0.61	0.52	0.13	24.82
	CI	0.64	0.61	0.60	0.21	1.19
Elevated blood pressure	BMI	0.72	0.57	0.60	0.16	21.1
	%BF	0.72	0.58	0.69	0.27	23.0
	WHR	0.71	0.60	0.60	0.20	0.85
	WHtR	0.71	0.52	0.68	0.20	0.47
	AVI	0.72	0.69	0.50	0.19	10.45
	BRI	0.72	0.52	0.68	0.20	2.82
	ABSI	0.68	0.64	0.51	0.15	0.08
	BAI	0.70	0.68	0.47	0.15	24.00
	CI	0.70	0.48	0.71	0.19	1.20
MetS by ATP	BMI	0.74	0.74	0.61	0.35	21.30
	%BF	0.74	0.74	0.63	0.37	23.10
	WHR	0.73	0.68	0.71	0.39	0.87
	WHtR	0.76	0.60	0.82	0.42	0.50
	AVI	0.77	0.63	0.78	0.40	12.94
	BRI	0.76	0.60	0.82	0.42	3.37
	ABSI	0.67	0.64	0.65	0.29	0.08
	BAI	0.68	0.73	0.55	0.28	24.82
	CI	0.72	0.64	0.74	0.37	1.21
MetS by IDF	BMI	0.93	0.94	0.73	0.67	22.40
	%BF	0.87	0.86	0.73	0.60	25.30
	WHR	0.94	0.95	0.82	0.77	0.90
	WHtR	0.97	0.99	0.89	0.88	0.53
	AVI	0.99	1.00	0.98	0.98	16.20
	BRI	0.97	0.99	0.89	0.88	3.86
	ABSI	0.80	0.84	0.66	0.50	0.08
	BAI	0.82	0.72	0.78	0.50	27.00
	CI	0.91	0.98	0.73	0.71	1.22

Body mass index (BMI); Body fat percentage (%BF); Waist circumference (WC); Waist–height ratio (WHtR); Abdominal Volume Index (AVI); Body roundness index (BRI); A body shape index (ABSI); Body adiposity index (BAI); Conicity index (CI).

**Table 4:** Areas under the curve (AUCs) and cut-off points for anthropometric indices for the prediction of MetS components in women.

MetS Components	Indices	AUC	Sensitivity	Specificity	Youden Index	Cut-Off Points
High triglycerides	BMI	0.72	0.76	0.51	0.27	20.73
	%BF	0.72	0.77	0.54	0.31	27.50
	WHR	0.69	0.75	0.52	0.27	0.80
	WHtR	0.72	0.68	0.64	0.32	0.48
	AVI	0.72	0.68	0.64	0.32	10.71
	BRI	0.72	0.68	0.64	0.32	2.94
	ABSI	0.64	0.63	0.55	0.17	0.08
	BAI	0.68	0.66	0.55	0.21	28.81
	CI	0.67	0.72	0.53	0.25	1.15
Low HDL-C	BMI	0.58	0.62	0.51	0.13	20.66
	%BF	0.57	0.48	0.63	0.11	29.30
	WHR	0.55	0.64	0.45	0.09	0.80
	WHtR	0.56	0.51	0.60	0.11	0.48
	AVI	0.56	0.72	0.38	0.10	9.39
	BRI	0.56	0.51	0.60	0.11	2.91
	ABSI	0.53	0.44	0.62	0.06	0.08
	BAI	0.54	0.48	0.58	0.06	29.29
	CI	0.53	0.58	0.50	0.08	1.14
Increased blood glucose	BMI	0.68	0.39	0.79	0.18	23.56
	%BF	0.68	0.57	0.69	0.26	30.90
	WHR	0.68	0.53	0.71	0.25	0.84
	WHtR	0.70	0.62	0.65	0.27	0.49
	AVI	0.70	0.46	0.81	0.27	12.39
	BRI	0.70	0.62	0.65	0.27	3.13
	ABSI	0.65	0.46	0.75	0.21	0.08
	BAI	0.67	0.57	0.62	0.19	29.70
	CI	0.67	0.52	0.73	0.25	1.20
Elevated blood pressure	BMI	0.79	0.49	0.72	0.21	22.27
	%BF	0.77	0.61	0.68	0.29	29.50
	WHR	0.77	0.61	0.61	0.22	0.82
	WHtR	0.78	0.66	0.64	0.30	0.47
	AVI	0.78	0.61	0.65	0.26	10.81
	BRI	0.78	0.66	0.64	0.30	2.90
	ABSI	0.76	0.61	0.54	0.15	0.08
	BAI	0.78	0.50	0.73	0.24	30.35
	CI	0.76	0.60	0.61	0.21	1.16
MetS by ATP	BMI	0.83	0.64	0.74	0.38	22.31
	%BF	0.81	0.68	0.77	0.45	30.90
	WHR	0.80	0.74	0.68	0.42	0.82
	WHtR	0.84	0.76	0.75	0.51	0.49
	AVI	0.85	0.59	0.89	0.48	12.37
	BRI	0.84	0.76	0.75	0.51	3.14
	ABSI	0.74	0.67	0.61	0.28	0.08
	BAI	0.79	0.67	0.66	0.34	29.61
	CI	0.79	0.60	0.80	0.40	1.21
MetS by IDF	BMI	0.90	0.73	0.84	0.58	23.58
	%BF	0.88	0.80	0.79	0.59	31.90
	WHR	0.90	0.87	0.80	0.67	0.85
	WHtR	0.96	0.95	0.87	0.82	0.51
	AVI	0.97	1.00	0.93	0.93	12.80
	BRI	0.96	0.95	0.87	0.82	3.60
	ABSI	0.81	0.78	0.69	0.47	0.08
	BAI	0.82	0.73	0.72	0.45	30.42
	CI	0.90	0.87	0.80	0.67	1.21

Body mass index (BMI); Body fat percentage (%BF); Waist circumference (WC); Waist–height ratio (WHtR); Abdominal Volume Index (AVI); Body roundness index (BRI); A body shape index (ABSI); Body adiposity index (BAI); Conicity index (CI).

**Table 5:** Relationship between the anthropometric indices with metabolic abnormalities in multivariate analysis.

Index	Men *		Women **	
	OR ^+^	95%CI	OR ^+^	95%CI
Body mass index (BMI)	4.10	3.18; 5.29	4.67	3.73; 5.84
Body fat percentage (%)	4.12	3.15; 5.38	4.31	3.45; 5.39
Waist–hip ratio (WHR)	3.80	2.97; 4.87	3.79	3.02; 4.75
Waist–height ratio (WHtR)	5.04	3.94; 6.45	6.14	4.87; 7.74
Abdominal Volume Index (AVI)	4.62	3.60; 5.93	6.82	4.15; 7.97
Body roundness index (BRI)	5.27	4.11; 6.75	6.25	4.95; 7.89
A body shape index (ABSI)	2.12	1.66; 2.70	2.05	1.63; 2.58
Body adiposity index (BAI)	2.76	2.15; 3.53	3.08	2.48; 3.83
Conicity index (CI)	3.57	2.79; 4.58	3.52	2.82; 4.41

* Cut-off points for anthropometric indices for the MetS diagnosis by NCEP ATP III in men: BMI—21.30; %BF—23.10; WHR—0.87; WHtR—0.50; AVI—12.94; BRI—3.37; ABSI—0.08; BAI—27.00; CI—1.22. ** Cut-off points for anthropometric indices for the MetS diagnosis by NCEP ATP III in women: BMI—22.31; %BF—30.90; WHR—0.82; WHtR—0.49; AVI—12.37; BRI—3.14; ABSI—0.08; BAI—29.61; CI—1.21. ^+^ Adjusted for age, living areas, ethnicity, smoking, alcohol use, and salty eating habits; reference groups were lower than cut-off point values. For all anthropometric indicators, reference groups are lower than the cut-off points.

## Data Availability

All data generated or analyzed during this study are included in this article and its Supplementary Material Files. Further inquiries can be directed to the corresponding author.

## Supplementary Materials

The following are available online at https://www.mdpi.com/article/10.3390/nu14194024/s1, Table S1: Socio-economic characteristics of participants.
