# Supplementary material for: Anthropometric Cut-Off Values for Detecting the Presence of Metabolic Syndrome and Its Multiple Components among Adults in Vietnam: The Role of Novel Indices
Source: Nutrients. 2022 Sep 28;14(19):4024. doi: 10.3390/nu14194024 (PMC9571833; doi:10.3390/nu14194024)
Supplement: Supplementary file 1 [file nutrients-14-04024-s001.zip › nutrients-1900067-supplementary.pdf]

Supplementary Materials

Table S1. Socio-economic characteristics of participants

	**Having MetS**		**Without MetS**		**Total**		***p*-value**
	**Frequency (*n*)**	**Percentage (%)**	**Frequency (*n*)**	**Percentage (%)**	**Frequency (*n*)**	**Percentage (%)**	
**Gender**							
Males	421	39.9	1856	50.9	2277	48.4	<0.01†
Females	635	60.1	1789	49.1	2424	51.6	
**Age (Mean, SD)**	55.2	12.3	48.0	14.2	49.6	14.1	<0.01*
**Age group**							
25-29	34	3.4	421	12.1	455	10.1	<0.01†
30-39	92	9.3	763	21.8	855	19.1	
40-49	210	21.3	801	22.9	1011	22.6	
50-59	282	28.6	701	20.1	984	22.0	
$\geq$ 60	369	37.4	807	23.1	1176	26.2	
**Living areas**							
Urban	151	14.3	1058	29.0	1209	25.7	<0.01†
Rural	207	19.6	973	26.7	1180	25.1	
Mountainous	272	25.8	915	25.1	1187	25.3	
Coastal	426	40.3	699	19.2	1125	23.9	
**Ethnic**							
Kinh	893	90.4	2985	85.0	3878	86.2	<0.01†
Others	95	9.6	526	15.0	621	13.8	
**Alcohol use**							
Never	461	46.7	1569	44.7	2030	45.1	0.20†
Special occasion	296	30.0	1111	31.6	1407	31.3	
Every week	104	10.5	321	9.1	425	9.5	

Every day	126	12.8	511	14.6	637	14.2	
**Smoking**							
Never	673	68.2	2252	64.1	2925	65.0	<0.01†
Sometimes/Rarely	47	4.8	244	7.0	291	6.5	
Always	266	27.0	1015	28.9	1281	28.5	
**Salty eating habits**							
Normal	436	44.2	1739	49.6	2175	48.4	<0.01†
Moderate	288	29.2	730	20.8	1018	22.6	
Extreme	263	26.6	1039	29.6	1302	29.0	

* T-test, † Chi-square test
